# Correlation between *Helicobacter pylori* Infection and Metabolic Abnormality in General Population: A Cross-Sectional Study

**DOI:** 10.1155/2018/7410801

**Published:** 2018-03-20

**Authors:** Li-juan Lu, Ning-Bo Hao, Jian-Jun Liu, Xue Li, Rui-Ling Wang

**Affiliations:** ^1^Department of Gastroenterology, General Hospital of the PLA Rocket Force, Beijing, China; ^2^Jinzhou Medical University, Jinzhou, China

## Abstract

**Background:**

Previous studies have suggested a link between *Helicobacter pylori* (*H. pylori*) and metabolic abnormality. This study aimed at investigating the correlation between *H. pylori* infection and metabolic abnormality in a general population.

**Methods:**

All enrolled participants underwent a carbon-13 urea breath test (^13^C-UBT). For each individual, the following data were collected: age, gender, alanine transaminase (ALT), total protein, albumin, cholesterol, triglyceride (TG), high-density lipoprotein cholesterol (HDL-C), low-density lipoprotein cholesterol (LDL-C), urea nitrogen, creatinine, uric acid, fasting plasma glucose, postprandial blood sugar, nonalcoholic fatty liver disease (NAFLD), and bone mineral density (BMD).

**Results:**

The study included 1867 (393 females and 1474 males, aged 54.0 ± 9.6 years) people that took a physical examination. There was no significant difference in gender and age between the study participants with and without *H. pylori* infection. The statistical data are as follows: albumin: *P* = 0.045, uric acid: *P* = 0.025, fasting glucose: *P* = 0.043, and postprandial blood glucose: *P* = 0.035. In terms of the patients with NAFLD, there were significant differences in ALT and HDL-C between the study participants with and without *H. pylori* infection. TG (*P* = 0.048), HDL-C (*P* = 0.011), and fasting blood glucose (*P* = 0.018) were significantly different in both groups among individuals who got osteopenia.

**Conclusion:**

*H. pylori* infection may be an important factor affecting metabolic abnormality and osteoporosis.

## 1. Introduction

*H. pylori*, a Gram-negative, spiral-shaped bacterium that dwells on the gastric epithelium, has an influence on approximately 50% of the human population, with a high rate in those living in developing countries. The prevalence of *H. pylori* infection is approximately 30% in developed countries and up to 80% in developing countries [1]. *H. pylori* infection induces chronic inflammation and immune responses in the stomach. *H. pylori* infection increases inflammatory factors, such as interleukin 1 (IL-1), interleukin 8 (IL-8), and tumor necrosis factor-alpha (TNF-*α*). These inflammatory factors may result in metabolic changes and systemic immune responses [2–4]. Moreover, *H. pylori* is the most prevalent pathogenic bacteria in the stomach and can lead to a wide array of gastric disorders, including chronic gastritis, peptic ulcer, gastric cancer, and gastric mucosa-associated lymphoid tissue lymphoma (MALT) [5]. It is also considered a class I carcinogen. In recent years, accumulating evidence has demonstrated the role of *H. pylori* infection in extragastrointestinal diseases, such as cardiovascular diseases, neurological disorders, metabolic diseases, and kidney disease [6–8]. IL-1 and TNF-*α* trigger bone resorption [9]. In addition, osteoporosis is a condition with progressively decreasing BMD and increased bone fragility, with increased risk of bone fractures [10]. Metabolic syndrome and osteoporosis have become worldwide public health issue, especially in elderly subjects, with huge clinical and economic burdens. Identifying risk factors with potential therapeutic implications is increasingly important in managing metabolic abnormality and osteoporosis and decreasing economic and health burdens.

This study aimed at analyzing the correlation between *H. pylori* infection and metabolic abnormality in middle-aged and the elderly general population.

## 2. Material and Method

This study was conducted at the General Hospital of PLA Rocket Force. All qualified subjects who attended their annual health examination during the year 2016 were initially enrolled.

### 2.1. Ethics

All subjects signed an informed consent prior to their enrollment in the study. Additionally, the study was planned according to the ethical guidelines following the declaration of the General Hospital of PLA Rocket Force.

### 2.2. Inclusion and Exclusion Criteria

Inclusion criteria were complete, having not used proton pump inhibitors (PPIs), histamine type 2 receptor antagonists (H_2_A), antibiotics, bismuth, or sucralfate for up to one month prior to the urea breath test.

Individuals who had received vitamin D supplementation, *H. pylori* eradication therapy, or antibiotics were excluded. Other exclusion criteria include self-reported history of chronic liver disease or cirrhosis, alcohol intake ≥ 30 g/day for men and 20 g/day for women, and positive serologic markers for hepatitis B or C virus. Participants with missing data on important covariates were excluded.

### 2.3. Method

In total, 1867 subjects who met these criteria were selected. All subjects who participated in a physical examination, from May 2016 to July 2016, were asked to take blood tests, a BMD examination, abdominal ultrasonography (US), and complete ^13^C-UBT for the diagnosis of active *H. pylori* infection. Biochemical investigation including ALT, total protein, albumin, cholesterol, TG, HDL-C, LDL-C, urea nitrogen, creatinine, uric acid, fasting plasma glucose, and postprandial blood sugar was performed in all individuals. After a 12 h overnight fast, 15 mL of blood was collected from the antecubital vein. It was analyzed within 4 hours after collection for biochemistry tests. After an overnight fast, ^13^C-UBT was performed using the Proto Pylori kit (Isodiagnostika Canada), containing 75 mg of ^13^C-UBT and additives. Two breath samples were collected within a 30-minute interval. Patient samples were analyzed by gas chromatography. The results were expressed as delta over baseline (DOB). BMD was measured by a quantitative ultrasound test (QUS, Sahara Clinical Bone Sonometer, HOLOGIC, Bedford, MA, USA) method in the calcaneus in all participants. Fatty liver was examined using a Philips HD 11 XE multifunction color Doppler diagnostic instrument, according to the standard criteria for NAFLD by the Chinese Hepatology Association dating from February 2006 [1]. All ultrasound measurements were performed by the same ultrasound physician throughout the study. The diagnostic criteria are as follows: ALT > 40 U/L, total protein > 5.69 mmol/L, albumin > 55 g/L, cholesterol > 5.69 mmol/L, TG > 1.7 mmol/L, HDL-C > 1.7 mmol/L, LDL-C < 3.64 mmol/L, urea nitrogen > 8.05 mmol/L, creatinine > 115 *μ*mol/L, uric acid > 416 *μ*mol/L, fasting plasma glucose > 6.1 mmol/L, and postprandial blood sugar > 7.8 mmol/L. Because each year the same population undergo for a physical examination in our hospital, we had access to information that could identify individual participants during or after data collection.

### 2.4. Statistical Analysis

Statistical analysis was performed using SPSS software version 17.0 (SPSS Inc., Chicago, IL). Continuous variables were checked by *t*-test, and the categorical variables were compared by a chi square test or Fisher exact probability method. Data are expressed as the mean and standard deviation. For all statistical analyses, the level of significance was considered to be *P* < 0.05.

## 3. Results

A total of 1867 participants (393 women and 1474 men) were included in this study. The characteristics of all individuals are presented in Table 1. The prevalence of *H. pylori* positivity was found to be 31.5% (589/1867). NAFLD was found to be 31.9% (596/1867). BMD deficiency was found in 48.2% of participants (900/1867).

The characteristics classified being *H. pylori*-positive or *H. pylori-*negative are shown in Table 2. It was observed that the individuals with high fasting plasma glucose had a higher rate *H. pylori* infection than those without *H. pylori* (13.8% versus 8.5%, *P* = 0.001). No difference was found between the two groups in terms of age or gender. In addition, fasting plasma glucose (5.36 ± 1.20 versus 5.25 ± 1.03, *P* = 0.043) and postprandial blood sugar (7.21 ± 2.50 versus 6.96 ± 2.29, *P* = 0.035) were significantly lower in the *H. pylori*-negative group, respectively.

In this study, participants with NAFLD had a higher rate of *H. pylori* infection than those without NAFLD (33.8% versus 31.1%). However, there were no statistically significant differences in NAFLD and *H. pylori* infection between the participants in this study (*P* = 0.241). Furthermore, we selected patients who were diagnosed with NAFLD. Those patients were divided into 2 groups according to *H. pylori* infection. Statistical results are shown in Table 3. The prevalence of NAFLD in the group with and without *H. pylori* were 199 patients (33.4%) and 397 patients (66.6%), respectively. Moreover, a significant difference was determined between the two groups in terms of gender (*P* = 0.009). Additionally, when *H. pylori*-positive and *H. pylori*-negative patients were compared, ALT (26.04 ± 15.68 versus 23.37 ± 13.42, *P* = 0.031) levels were found to be significantly higher in *H. pylori*-positive patients; however, HDL-C (1.12 ± 0.26 versus 1.19 ± 0.28, *P* = 0.005) levels were found to be lower with *H. pylori* infection.

Finally, the patients who were diagnosed with osteoporosis were picked up. Similarly, those patients were divided into 2 groups based on *H. pylori* infection. The results are shown in Table 4. We could clearly find that the prevalence of low HDL-C in the group with and without *H. pylori* was 54 patients (18.8%) and 76 patients (12.4%), respectively, in which this difference was significant (*P* = 0.011). Fasting plasma glucose, in this cohort, also showed a statistically significant difference (*P* = 0.018). According to the TG levels, there was a significant difference in the 2 groups (1.54 ± 1.01 versus 1.40 ± 0.80, *P* = 0.048).

## 4. Discussion

*H. pylori*, colonized in the human stomach, is an ancient organism that has coevolved with humans for many years [11]. In 1982, Warren and Marshall cultivated the bacterium from mucosal layer of stomach [12]. After three decades, *H. pylori* infection has an influence on approximately 50% of humans, especially those living in developing countries, which is up to 80%. In this study, the overall infection rate was 31.5%. The infection rate was relatively lower than average infection rates. Since the included human beings were in highly socioeconomic populations, it is widely acknowledged that the incidence of infection is strongly influenced by socioeconomic factors [13, 14]. Risk factors for *H. pylori* infection are related to living in crowded conditions, living without a reliable supply of clean water, living in a developing country, and living with someone who has a *H. pylori* infection [15].

NAFLD is a common disease that affects 25%–30% of the population in western countries [16]. NAFLD is more often diagnosed among patients with *H. pylori* [17]. According to previous investigation, *H. pylori* infection may be one of the factors that contributes to the pathogenesis of NAFLD. So eradicating *H. pylori* may be significant in the treatment of this disease [18]. However, the pathogenic mechanism of this relationship is unclear. A multitude of previous reports concluded that the infection of *H. pylori* is associated with an altered serum lipid profile. It is also considered a risk factor for atherosclerosis and NAFLD [2, 19]. *H. pylori* may lead to liver injury via specific toxins [20]. In addition, invasion of *H. pylori* in the small bowel mucosa might increase gut permeability and facilitate the passage of bacterial endotoxins via the portal vein to the liver [21]. Previous studies have suggested that the association between *H. pylori* infection and NAFLD is controversial. Kim et al. found that patients with *H. pylori* infection were at higher risk of NAFLD development compared to an uninfected individual [2]. Another study of 13,737 Japanese adults reported that *H. pylori* infection is not associated with NAFLD [22]. These findings are in favor with the second result. When patients with NAFLD alone were studied, dividing them based on *H. pylori* infection, gender, and HDL-C had significantly different results. This finding indicates that protective factors for NAFLD were HDL-C and female gender. These results may need to be confirmed in multicenter and prospectively designed studies. The previous studies showed that *H. pylori* infection had a positive association with high LDL-C and low HDL-C [16, 23]. Similarly, this research agrees with this view.

It is considered that *H. pylori* infection plays a pathophysiologic role in several endocrine disorders. Numerous studies found an association between *H. pylori* infection and diabetes [24, 25]. Nevertheless, there is no agreement among researchers in this regard. A meta-analysis study showed that there was insufficient evidence that *H. pylori* infection worsened glycemic control in patients with diabetes [26]. However, another meta-analysis investigation found that *H. pylori* infection was more frequent in diabetic patients [27]. The mechanism of this relationship is still unclear. In this study, *H. pylori* infection was etiologically associated with fasting plasma glucose and postprandial blood sugar. Several studies examined the associated between *H. pylori* infection and insulin resistance (IR) [28]. Jeon et al. proposed the possible role of altered gut microbiota in the pathogenesis of insulin resistance and diabetes [29]. These findings were in agreement with the results that *H. pylori* infection was associated with diabetes. However, the results of annual health examination may differ from the patients' true blood glucose levels. Further examination of patients' glycated hemoglobin (HbA1c) was needed to elucidate the association between *H. pylori* infection and diabetes.

Osteoporosis poses a great threat to the elderly population. It is a major cause of bone fracture and morbidity, disability, and mortality in elderly individuals, affecting their families and the health care system [30]. *H. pylori* infection could lead to chronic local and systemic immune response. The resulting increase in proinflammatory cytokines could affect bone resorption and might increase the risk of osteoporosis system [31]. The risk factors for osteoporosis include cigarette smoking, excessive alcohol consumption, vitamin D deficiency, and low dietary calcium [32]. Calcium is ionized in acidic conditions and absorbed in the small bowel. Therefore, in either hypochlorhydric or achlorhydric stomachs, calcium absorption is impaired [33]. Conditions causing a decrease in gastric acid secretion status included gastric surgery, use of PPIs, and infection with *H. pylori* [32, 34, 35]. *H. pylori* infection causes chronic gastritis and induces a permanent inflammatory response that may increase both the gastric and the systemic indexes of inflammation, such as TNF-*α*, IL-1, and IL-6 [35]. Shih et al. demonstrated that *H. pylori* infection may be associated with an increased risk of developing osteoporosis in elderly female patients with upper gastrointestinal diseases [36]. Meantime, Figura et al. found that *H. pylori* infection by strains expressing CagA may be considered a risk factor for osteoporosis in men [35]. In contrast, Fotouk-Kiai et al. revealed that *H. pylori* infection is not associated with BMD changes in the elderly population [37]. Similarly, in this study, it was not revealed that there was a difference of the prevalence of osteoporosis in *H. pylori*-positive and *H. pylori*-negative groups. Maybe it was caused by the relatively low included general population. Moreover, patients with osteoporosis were studied, dividing them based on *H. pylori* infection. Like other subgroups, we also demonstrated that HDL-C was associated with *H. pylori* infection (*P* = 0.011). In addition, according to fasting plasma glucose, there was a significant difference in this subgroup. Jackuliak and Payer revealed that patients with diabetes mellitus have an increased risk of bone fractures [38]. In contrast, Kumar et al. found that the use of oral hypoglycemic agents for a period of three years or more does not significantly affect the BMD in patients with diabetes mellitus [39]. Apparently, this study was in favor with the first conclusion. When we analyzed subgroups of patients with osteoporosis and NAFLD, *H. pylori* infection had a positive association with low HDL-C and high TG.

The evaluation of an association between *H. pylori* infection and causes of chronic renal failure was limited [40]. A recent meta-analysis study demonstrated that end-stage renal disease could be a potential protective factor for *H. pylori* infection [41]. However, Gu et al. found no evidence of a significant association between *H. pylori* infection and dialysis overall, whereas long-term treatments of more than four years had a significant protective effect [42]. Maybe, patients with long-term dialysis were not required to eradicate *H. pylori*. In this study, we revealed that *H. pylori* infection was in association with uric acid from Table 3.

A few limitations warrant consideration. First, the average age of our study population was 54.0 ± 9.6 years. This might be due to the presence of more risk factors for the development of metabolic abnormalities in people who are in this age group. Second, a number of studies have demonstrated that *H. pylori* infection by strains expressing CagA can significantly induce gastric cancer [43]. However, in this study, the included general population were in a physical examination, and the CagA status was not detected. In the further research, we will discuss the role of *H. pylori* infection by strains expressing CagA in metabolic abnormality. Third, other factors, such as being overweight, abdominal circumference, gene, and diet, may affect *H. pylori* inflammation and regulation of glycemic index. Fourth, this study was only a single-center study. Therefore, data collection from young general population and multicenter studies are necessary for future studies.

In summary, it was found that *H. pylori* infection may be an important factor affecting metabolic abnormality and osteoporosis. This indicates that *H. pylori* eradication might play a significant role in reducing the risk of metabolic abnormality and osteoporosis.

## Figures and Tables

**Table 1:** Characteristics of all participants.

Number (women/man)	393/1474
Age	54.0 ± 9.6
ALT	19.9 ± 12.7
Total protein	72.5 ± 3.7
Albumin	46.0 ± 2.3
Cholesterol	4.7 ± 0.9
TG	1.5 ± 0.9
HDL-C	1.3 ± 0.4
LDL-C	3.1 ± 0.8
Urea nitrogen	5.3 ± 1.3
Creatinine	79.0 ± 15.1
Uric acid	334.4 ± 78.3
Fasting plasma glucose	5.3 ± 1.1
Postprandial blood sugar	7.0 ± 2.4
*H. pylori* positive	589 (31.5%)
NAFLD	596 (31.9%)
BMD	900 (48.2%)

**Table 2:** Characteristics of categorical variables of all participants based on *H. pylori* infection.

Variables	*H. pylori* (+)	*H. pylori* (−)	*P* value
Number (women/man)	114/475	279/999	0.223
BMD	287 (48.7%)	613 (48.0%)	0.765
NAFLD	199 (33.8%)	397 (31.1%)	0.241
ALT	35 (5.9%)	58 (4.5%)	0.195
Cholesterol	74 (12.6%)	134 (10.5%)	0.185
TG	161 (27.3%)	314 (24.6%)	0.202
HDL-C	103 (17.5%)	194 (15.2%)	0.205
LDL-C	145 (24.6%)	320 (25.0%)	0.845
Creatinine	8 (1.4%)	14 (1.1%)	0.625
Urea nitrogen	20 (3.4%)	28 (2.2%)	0.126
Uric acid	99 (16.8%)	174 (13.6%)	0.070
Fasting plasma glucose	81 (13.8%)	109 (8.5%)	0.001^※^
Postprandial blood sugar	142 (24.1%)	287 (22.5%)	0.430

※ represents *P* value less than 0.05 between *H. pylori*-positive and *H. pylori*-negative groups.

**Table 3:** Characteristics of categorical variables of patients diagnosed as nonalcoholic fatty liver disease (NAFLD) on *H. pylori* infection.

Variables	*H. pylori* (+)	*H. pylori* (−)	*P* value
Number (women/man)	17/182	65/332	0.009^※^
BMD	111 (56.9%)	193 (49.5%)	0.090
ALT	19 (9.5%)	33 (8.3%)	0.926
Cholesterol	29 (14.6%)	51 (12.8%)	0.560
TG	94 (47.2%)	171 (43.1%)	0.335
HDL-C	68 (34.2%)	100 (25.2%)	0.022^※^
LDL-C	57 (28.6%)	110 (27.7%)	0.810
Creatinine	3 (1.5%)	7 (1.8%)	0.819
Urea nitrogen	4 (2.0%)	9 (2.3%)	0.840
Uric acid	49 (24.6%)	79 (19.9%)	0.185
Fasting plasma glucose	40 (20.1%)	64 (16.1%)	0.227
Postprandial blood sugar	63 (31.7%)	135 (34.0%)	0.566

※ represents *P* value less than 0.05 between *H. pylori*-positive and *H. pylori*-negative groups.

**Table 4:** Characteristics of categorical variables of patients diagnosed as osteoporosis based on *H. pylori* infection.

Variables	*H. pylori* (+)	*H. pylori* (−)	*P* value
Number (women/man)	57/230	151/462	0.119
ALT	20 (7.0%)	29 (4.7%)	0.168
Cholesterol	41 (14.3%)	67 (10.9%)	0.149
TG	82 (28.6%)	149 (24.3%)	0.172
HDL-C	54 (18.8%)	76 (12.4%)	0.011^※^
LDL-C	75 (26.1%)	166 (27.1%)	0.765
Urea nitrogen	14 (4.9%)	15 (2.4%)	0.054
Creatinine	5 (1.7%)	9 (1.5%)	0.757
Uric acid	44 (15.3%)	80 (13.1%)	0.355
Fasting plasma glucose	43 (15.0%)	59 (9.6%)	0.018^※^
Postprandial blood sugar	70 (24.4%)	156 (25.4%)	0.733

※ represents *P* value less than 0.05 between *H. pylori*-positive and *H. pylori*-negative groups.
